# Periodontal effects of maxillary expansion in adults using non-surgical expanders with skeletal anchorage vs. surgically assisted maxillary expansion: a systematic review

**DOI:** 10.1186/s13005-021-00299-7

**Published:** 2021-11-10

**Authors:** José Antonio Vidalón, Ismael Loú-Gómez, Aldo Quiñe, Karla T. Diaz, Carlos Liñan Duran, Manuel O. Lagravère

**Affiliations:** 1 Department of Orthodontics, Faculty of Stomatology, Universidad Peruana Cayetano Heredia, Lima, Peru; 2 Stomatology Second Speciality, Universidad Privada San Juan Bautista, Lima, Peru; 3 University of Alberta, Faculty of Medicine and Dentistry, School of Dentistry, Orthodontic Graduate Program, ECHA 5-524, 11405-87 Ave, Edmonton, AB T6G 1C9 Canada

**Keywords:** Maxillary expansion, Palatal, Periodontal, Adult, Tomography

## Abstract

**Objectives:**

Describe and compare harmful periodontal effects as a consequence of maxillary expansion in adult patients with different types of anchorage devices in non-surgical expanders with skeletal anchorage and surgically assisted maxillary expansion.

**Materials and methods:**

An exhaustive search was carried out on the electronic databases PubMed (MEDLINE), Embase, Cochrane and LILACS. Additionally, journal references and grey literature were searched without any restrictions. After the selection and extraction process; risk of bias was assessed by the ROB-1 Cochrane tool and Newcastle-Ottawa Scale (NOS) for randomized trials and cohort studies, respectively.

**Results:**

Of 621 studies retrieved from the searches, six were finally included in this review. One of them presented a low risk bias, while five were excellent respective to selection, comparability and outcomes. Results showed that maxillary expansion in adults using non-surgical expanders (bone-borne or tooth-bone-borne with bicortical skeletal anchorage) produce less harmful periodontal effects, such as: alveolar bending with an average range from 0.92° to 2.32°, compared to surgically assisted maxillary expansion (tooth-borne) of 6.4°; dental inclination with an average range from 0.07° to 2.4°, compared to surgically assisted maxillary expansion (tooth-borne) with a range from 2.01° to 5.56°.

**Conclusions:**

Although limited, the current evidence seems to show that the bone-borne or tooth-bone-borne with bicortical skeletal anchorage produces fewer undesirable periodontal effects.

**Supplementary Information:**

The online version contains supplementary material available at 10.1186/s13005-021-00299-7.

## Introduction

Non-surgical rapid maxillary expansion (RME) has been used to treat transversal deficiencies of the maxilla for adolescents and children [1, 2]. However, in adult patients RME is rarely successful due to the fact that the palatal suture and the adjacent joints begin to fuse at the end of adolescence and become more rigid with age [3]. Adverse effects of RME have been reported in adults, such as: instability of results, pain, edema,, gingival recession, root resorption, ulceration of the palate mucosa, tooth inclination and alveolar bending [3, 4] Although, in general RME is recognized as a safe and reliable treatment, it causes lateral flexion of the alveolar processes (alveolar bending), because the points of application of the transverse force are positioned much lower than the centers of resistance of the maxillary halves. The same is true for the anchorage teeth because the point of force application is positioned lower than the center of resistance of the anchorage teeth, producing a buccal tipping or dental inclination of the involved teeth [5, 6].

The alternative for these patients is surgically assisted maxillary expansion (SARME). The surgery consists of osteotomies of the lateral walls of the maxilla and pterygoid plates, as well as release of the nasal septum, that are the main structures of resistance for maxillary expansion in adults. Unfortunately, adult patients tend to decline surgery [7, 8].

Lee et al. [9] and Wilmes B et al. [1] proposed a hybrid expander with skeletal anchorage (miniscrew-assisted rapid palatal expansion-MARPE) to prevent the undesirable effects of conventional RME in adults and to avoid the need for surgery to release the fused sutures. The intent of their proposal was to corroborate the effective separation of the palatal suture in adult patients with little buccal inclination of the anchored teeth.

Currently, therapeutic approaches for the correction of transverse problems in adult patients include MARPE or SARME. However, both procedures store residual forces produced by the expander device which are transmitted to the anchored teeth and then to the periodontal tissues [10, 11]. During maxillary expansion, these residual forces produce compression in the periodontal ligament on the buccal surfaces of the teeth, reducing the thickness of the buccal bone and inducing the formation of dehiscence and vertical alveolar bone loss in adults [12].

Three-dimensional investigations demonstrated that conventional RME induces a highly variable individual response, and the expansion force causes unwanted tooth movement thereby harming the periodontal tissues and, in some cases, causing defects [13, 14]. These have been reported in children and adolescents where the suture is still able to be split non-surgically. Thus, in adults, where the suture is more interdigitated, it is expected that the side effects on the periodontium can be more severe [15]. Some investigators have shown strong correlations between periodontal effects and RME [13, 14]. However, there are different types of anchorage devices for maxillary expansion (bone-borne, tooth-bone-borne and tooth-borne), with different periodontal effects in adult patients having non-surgical or surgical maxillary expansion.

The aim of this systematic review was to describe and compare the possible periodontal effects as a consequence of maxillary expansion with different types of anchorage devices in non-surgical expanders with skeletal anchorage and surgically assisted maxillary expansion.

## Materials and methods

### Registration and development

The systematic review protocol was registered in PROSPERO (International database of prospectively registered systematic reviews in health) on July, 14, 2020 under CRD XXXXXXXX (https:// www.crd.york.ac.uk/PROSPERO/), and developed in accordance with the PRISMA (Preferred Reporting Items for Systematic Reviews and Meta-Analyses) check-list of systematic reviews and meta-analyses [16].

### Search strategy

A detailed search in the main databases (PubMed, Medline, Cochrane Library and LILACS) was carried out using strategies based on the thesaurus and free term combinations associated with the research question (Additional file 1). Complementary, high impact clinical journals, available grey literature (clinicaltrials.gov, open grey and Google Scholar) and references of included studies were searched. The search included studies up to May 2020. No date or language restrictions were applied. The search was updated in all electronic databases up to April 2021 finding no additional studies.

### Selection of studies

An excel sheet was created for the selection process. All references from each database were transcribed and duplicates removed. The articles were triaged by title, abstract and full text independently by the first two authors (xx, xx). Disagreements were resolved through a consensus meeting and consultation with a third author (xx), if necessary.

#### Eligibility criteria

The eligibility criteria were defined based on the PICOT research strategy for clinical practice based on scientific evidence:

### Inclusion criteria

1. Participants: Adults patients (subjects over 18 years old) treated with maxillary expansion.

2. Intervention: Non-surgical bone-borne and tooth-bone-borne anchorage expanders (C-Expander, Maxillary Skeletal Expander-MSE and Hybrid Hyrax).

3. Comparison: Surgically assisted expansion with tooth-borne and tooth-bone-borne anchorage (Hyrax and hybrid hyrax).

4. Outcome: Periodontal effects: alveolar bending, tooth inclination, crest level height loss, alveolar bone thickness, fenestration and dehiscence.

5. Types of studies: Randomized, non-randomized, prospective, or retrospective clinical trials.

### Exclusion Criteria

1. Studies reporting patients with cleft lip and or palate or any craniofacial anomalies.

2. Any study that did not follow the PICOT criteria.

3. Case series, opinion articles, in vitro or animal studies, and literature reviews.

### Data collection

The data extraction table included the following information: principal author’s name, year of publication, sample size, age, expansion method, maxillary expander device, anchorage type, activation protocol, cone beam computed tomography (CBCT) settings, observation period, periodontal outcomes reported and measurable outcome results. This phase was also developed independently by the first two authors (xx, xx), with a consensus meeting and a third author opinion (xx) if necessary.

### Risk of Bias

The risk of bias tool, version 1, from the Cochrane Collaboration was used for randomized control trials [17], evaluation of random sequence generation, allocation concealment, blinding of participants and personnel, blinding of outcome assessment, incomplete outcome data and selective reporting bias. Assessment was carried out using red, yellow and green icons for high, unclear and low risk of bias respectively.

The Newcastle-Ottawa Scale (NOS) [18] was applied for retrospective cohort studies. Domains were evaluated with respect to selection of the cohort, confounders and outcomes. Each possible response within domains has a rating (star). The final star count given to each study can be interpreted as: 0–3 = poor; > 3–6 = fair; > 6–8 = good; and > 8–9 = excellent study.

The authors (xx, xx) were previously calibrated in rounds about tool use (xx), achieving a positive kappa value of 0.9. If any disagreement was found, items were discussed until consensus was achieved or another author made the final decision (xx).

## Results

### Studies selection

**Fig. 1:**
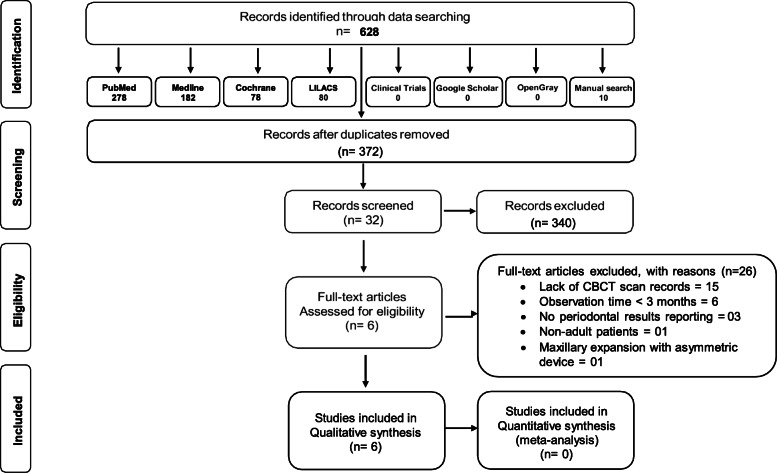
Flow chart of selected studies

A total of 621 references were found through electronic and manual searches and then duplicates were removed and selection processes were developed (Fig. 1). After title and abstract screening, 32 potential studies remained for full text reading. Among them, 26 were excluded due to lack of CBCT scan records, observation time < 3 months, no reporting of periodontal results, non-adult patients and maxillary expansion with asymmetric devices. Finally, only 6 articles were included for the qualitative analysis [19–24].

### Clinical results

A summary of the selected articles and results are shown in Table 1.

**Table 1:**
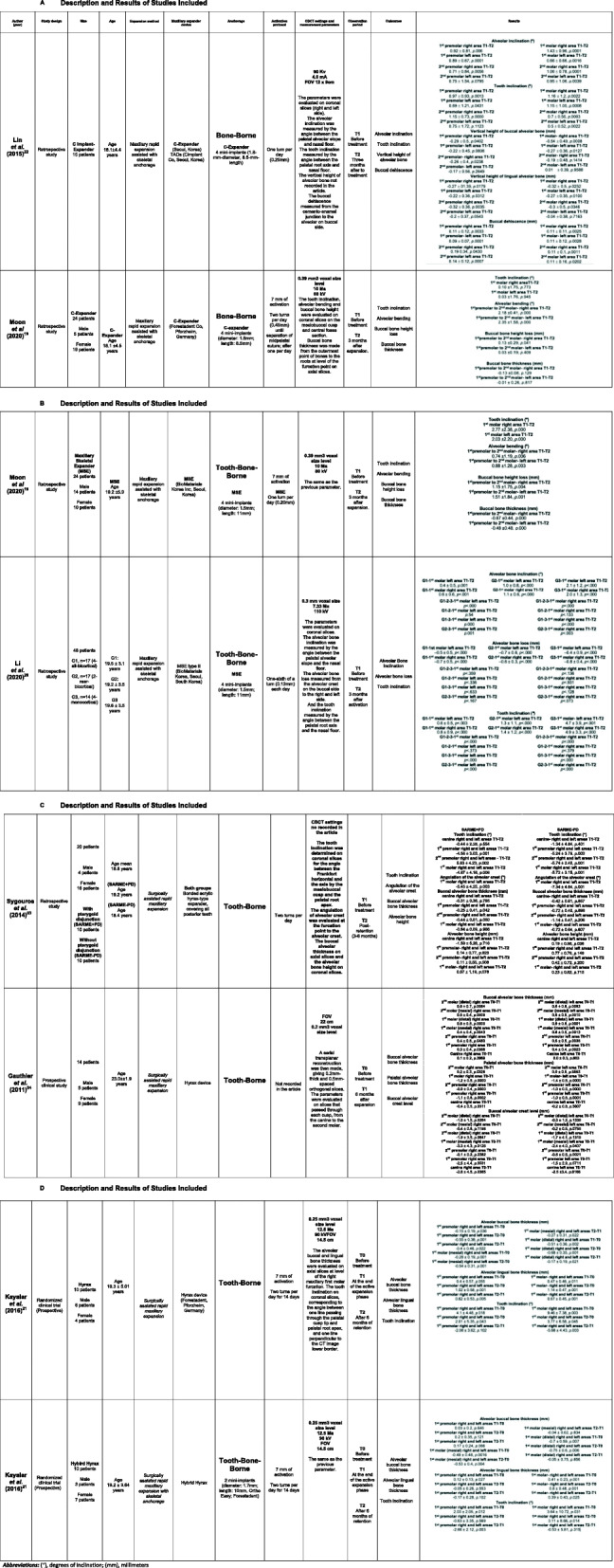
Description and Results of Studies Included

### Alveolar bending

Two studies [19, 22] in MARPE bone-borne reported 0.92° to 2.32°; two studies [19, 20] in MARPE tooth-bone-borne reported 0.5° to 2.05°, while only one study [23] in SARME tooth-borne reported an average 6.4° of alveolar inclination, *P* < .05.

### Dental inclination

Two studies [19, 22] in MARPE bone-borne reported 0.07° to 0.87°; two studies [19, 20] in MARPE tooth bone-borne reported 0.7° to 4.8°; two studies [21, 23] in SARME tooth-borne reported 2.01° to 5.56°; and one study [21] in SARME tooth-bone-borne reported − 0.63° to 3.11° of dental inclination, *P* < .05.

### Alveolar crest height

Two studies [19, 22] in MARPE bone-borne reported − 0.24 mm to − 1.24 mm; two studies [19, 20] in MARPE tooth-bone-borne reported − 0.6° to − 1.33°; and two studies [23, 24] in SARME tooth-borne, reported a range of − 0.31 mm to − 1.42 mm of decrease in alveolar crest height, *P* < .05.

### Buccal alveolar bone thickness

Moon et al. [19] reported − 0.07 mm in MARPE bone-borne and − 0.58 mm with tooth bone-borne; three studies [21, 23, 24] in SARME tooth-borne reported − 0.51 mm to − 0.86 mm and one study [21] in SARME tooth-bone-borne reported − 0.2 to − 0.64 mm of decrease in buccal alveolar thickness, *P* < .05.

### Palatal alveolar bone thickness

Two studies [21, 24] in SARME tooth-borne reported 0.85 mm to 1.14 mm and one study [21] in SARME tooth-bone-borne reported − 0.05 to 0.8 mm palatal alveolar bone thickness increases, *P* < .05.

### Dehiscence

Two studies in MARPE bone-borne reported 0.22mm^21^ (*P* < .05) and an incidence of 4.2% (2/48 cases) [22] and one study [19] in MARPE tooth-bone borne reported an incidence of 31.3% (15/48 cases) [19].

### Fenestration

Moon et al. [19] reported fenestrations in 1/48 cases in MARPE bone-borne and 6/48 cases with MARPE tooth-bone-borne.

### Methodological analysis

#### Risk of bias for randomized controlled trials

Only one study [21] was evaluated using the Cochrane ROB-1 tool (Table 2). Sequence generation, allocation concealment, selective outcome reporting and other bias domains were satisfactorily evaluated. Information about blinding of participants, personnel and outcome assessment were not described. Although it is mentioned that there were no harmful events, neither the results nor the final data confirm that the number of participants included at the beginning of the study was maintained. Attempts to contact the article author by email were not successful. Overall this study has a predominantly low risk bias (Table 3).

**Table 2:**
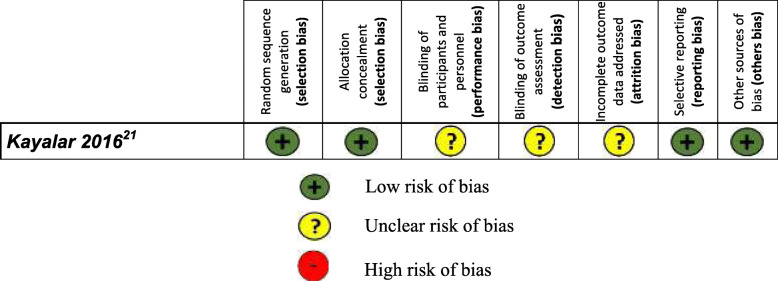
Risk of bias for randomized controlled trials

**Table 3:** ROB-1 Cochrane evaluation

DOMAIN	SELECTION BIAS				PERFORMANCE BIAS		DETECTION BIAS		ATTRITION BIAS		REPORTING BIAS		OTHER BIAS	
ITEM	***Sequence generation***		***Allocation concealment***		***Blinding of participants and personnel***		***Blinding of outcome assessment***		***Incomplete outcome data***		***Selective outcome reporting***		***Other sources of bias***	
**Author, year**	**Evaluation**	**Valuation support**	**Evaluation**	**Valuation support**	**Evaluation**	**Valuation support**	**Evaluation**	**Valuation support**	**Evaluation**	**Valuation support**	**Evaluation**	**Valuation support**	**Evaluation**	**Valuation support**
**Kayalar 201 6**[21]	**Low risk**	Randomization was performed using computer-generated tables.	**Low risk**	Allocation was concealed using sequentially numbered opaque and sealed envelopes.	**Unclear risk**	Although blinding was probably not possible, author does not mention information on this topic anywhere	**Unclear risk**	Author does not mention information on this topic anywhere	**Unclear risk**	No serious adverse effects were observed but no information about participant continuation or losses was reported	**Low risk**	Principal study outcomes were found in results section (skeletal expansion, dental expansion, periodontal side effects (dental tipping, root resorption, and buccal bone resorption)	**Low risk**	The study appears to be free of other sources of bias

#### Newcastle-Ottawa scale for cohort studies

The authors considered that all fives studies [19, 20, 22–24] had a representative and sufficient enough sample. Due to the complexity of these orthodontic procedures, it is very difficult to have a larger number of participants. All sample groups came from the same population of interest but only two studies [22, 23] detailed how sample size was calculated according to selection criteria. Surgical procedures and the handling of expansion devices were recorded and described with each author’s own variations. No study showed expansion before orthodontic management. Confounding factors such as age (principal), gender and periodontal health were controlled. A unique study [24] mentioned an independent blind assessment, in others [19, 22] tomographic evaluations were taken from computer records, which presumes an automatic generation of record linkage for each patient and is an acceptable outcome assessment. Finally, all studies reported an adequate follow-up time with no loss among the treated individuals (Table 4). As an overall result, all the studies evaluated with NOS had a score of 9 stars which is considered excellent.

**Table 4:** Newcastle-Ottawa scale for cohort studies

Newcastle-Ottawa scale for cohort studies		Selection of the cohort													Confounder		Cohort outcome										Score
		1) Representativeness of the exposed group/cohort				2) Selection of the non-exposed group/cohort			3) Ascertainment of exposure				4) Demonstration that outcome of interest was not present at start of study		5) Comparability of groups on the basis of the design or analysis		6) Assessment of outcome				7) Was Follow-Up Long Enough for Outcomes to Occur		8) Adequacy of Follow Up of Cohorts				
***Author, year***	***Type of study***	truly representative of the average (describe) in the community *	somewhat representative of the average in the community *	selected group of users	no description of the derivation of the cohort	drawn from the same community as the exposed cohort *	drawn from a different source	no description of the derivation of the non exposed cohort	secure record (eg surgical records) *	structured interview *	written self report	no description	Yes*	No	study controls for (select the most important factor) *-ex age	study controls for any additional factor * (This criteria could be modified to indicate specific control for a second important factor.)	independent blind assessment *	record linkage *	self report	no description	yes (select an adequate follow up period for outcome of interest) *	No	Complete follow up - all subjects accounted for *	subjects lost to follow up unlikely to introduce bias - small number lost - > ___ % (select an adequate %) follow up, or description of those lost) *	follow up rate < ___% (select an adequate %) and no description of those lost	No statement	
**Gauthier et al, 201 1**[24]	Prospective Cohort		*			*			*				*		*	*	*				*		*				*********
**Li et al, 202 0**[20]	Retrospective Cohort		*			*			*				*		*	*		*			*		*				*********
**Lin et al, 201 5**[22]	Retrospective Cohort		*			*			*				*		*	*		*			*		*				*********
**Moon et al, 202 0**[19]	Retrospective Cohort		*			*			*				*		*	*		*			*		*				*********
**Sygouros et al, 201 4**[23]	Retrospective Cohort		*			*			*				*		*	*		*			*		*				*********

## Discussion

Transverse skeletal deficiency in adults can be treated with SARME or with MARPE. Due to the increase in skeletal resistance in adults, maxillary expansion has been associated with harmful periodontal effects [19–26].

This study presents a descriptive comparison of the periodontal effects associated with SARME and MARPE. After the selection process, a total of 6 publications with a global sample of 165 patients were included. These studies reported quantitative measurements of the different periodontal indicators. This quantitative analysis was carried out to evaluate the periodontal effects before and after 3 or 6 months of maxillary expansion (Table 5), evaluating dental inclination, alveolar bending, buccal and palatal alveolar thickness, dehiscence and fenestrations.

**Table 5:** Comparison between Periodontal effects outcomes of MARPE and SARME

Periodontal outcomes	MARPE						SARME				
	Lin^**22**^ (between 1st PM to 2nd M)	Moon^**19**^ (1st M)		Li^**20**^ (1st M)			Sygourus^**23**^ (between 1st PM to 1st M)		Gauthier^**24**^ (between 1st PM to 2nd M)	Kayalar^**21**^ (1st PM/1 M)	
	Bone-Borne	Bone-Borne	Tooth-Bone-Borne	Tooth-Bone-Borne			Tooth-Borne		Tooth Borne	Tooth-Borne	Tooth-Bone-Borne
	C-Expander	C-Expander	MSE	G1 (MSE II) Bicortical	G2 (MSE II) Monocortical and bicortical	G3 (MSE II) Monocortical	+PD Hyrax-Acrylic	-PD Hyrax-Acrylic	Hyrax	Hyrax	Hybrid Hyrax
**Alveolar Bending**	0.92°	2.32°	0.81°	0.5°	1.05°	2.05°	5.45°	7.34°	–	–	–
**Dental inclination**	0.87°	0.07°	2.4°	0.7°	1.35°	4.8	5.03°	5.56°	–	2.01°/3.77°	−0.63°/3.11°
**Alveolar crest height**	−0.24 mm	−1.24 mm	−1.33 mm	−0.6 mm	−0.65 mm	−0.6 mm	0.31 mm	0.47 mm	−1.42 mm	–	–
**Buccal alveolar bone thickness**	–	−0.07 mm	− 0.58 mm	–	–	–	− 0.51 mm	− 0.86 mm	− 0.55 mm	− 0.55 mm/− 0.61 mm	− 0.2 mm/− 0.64 mm
**Palatal Alveolar bone thickness**	–	–	–	–	–	–	–	–	0.85 mm	1.02 mm/1.14 mm	− 0.05 mm/0.8 mm
**Dehiscence**	0.22 mm	2 of 48 (4.2%)	15 of 48 (31.3%)	–	–	–	–	–	–	–	–
**Fenestration**	–	1 of 48	6 of 48	–	–	–	–	–	–	–	–

*Abbreviations*: *1st PM* First premolar, *1st M* First molar, *+PD* With pterygoid disjunction, −PD Without pterygoid disjunction

Methodologically, all the included studies had an acceptable risk of bias. Due to the heterogeneity of the publications included in relation to the periodontal effects and the different methodologies used, the preparation of a meta-analysis was not justified and would not have allowed appropriate comparisons.

Only one study [24], mentioned blinding in measurements in CBCT images and two studies [19, 22] mentioned that it was impossible to measure the data using blinding methods because the appliances were inevitably shown in the CBCT images. While it is true, the absence of a blinding report may have been due to the inability to perform blinding because of visual characteristics of the orthodontic appliances.

Systematic reviews suggest that CBCTs allow for visualization of the periodontal tissues with accuracy (dento-alveolar structures) but offer poor contrast in soft tissues, improves diagnosis and optimizes treatment plans. However, its high radiation dose and cost-benefit ratio should be carefully analyzed before using it in periodontal diagnosis. This was not the case in the present study where the periodontal effects of SARME and MARPE were analyzed [25–27].

The periodontal effects in MARPE or SARME appear to depend on the type of anchorage used by the expander device. In this way, the alveolar inclination is less using MARPE tooth-bone-borne with bicortical anchorage (MSE II, G1 = 0.5°) [20], activated one sixth of a turn per day. It is slightly higher using MARPE bone-borne (C-expander = 0.92°, activated once per day) [22], and (C-expander = 2.32°, activated twice per day) [19].

The periodontal effects are significantly higher using SARME tooth-borne with or without pterygoid disjunction (hyrax = 6.4° on average of alveolar inclination) [23], activated 8 turns intraoperatively and after a latency period of 3 days, twice per day.

Everything indicates that the bicortical anchorage (cortical of the palatal bone and nasal floor) transmits the forces generated by the activation of the expander device directly to the bone, minimizing the alveolar inclination.

The remodeling of the alveolar process, due to the dental inclination produced by RME, could influence the dentoalveolar width of the maxilla, which is considered a determining factor of relapse [28].

Moon et al. [19] and Lin et al. [22] reported less dental inclination using MARPE bone-borne with C-expander = 0.07° and 0.87°, respectively, and MARPE tooth-bone-borne with bicortical anchorage (MSE II: G1 = 0.7 ° and G2 = 1.35°) [20]. The dental inclination is significantly higher using MARPE tooth-bone-borne monocortical (MSE II: G3 = 4.8°) [20], or SARME tooth-borne. According to Sygouros et al. [23] and Kalayar et al. [21], the level of the first molar was 5.4° and 3.77°, respectively. The periodontal effects were slightly attenuated in SARME tooth-bone-borne [21] with hybrid hyrax = 3.11°. To minimize dental inclination, we should choose a MARPE bone-borne or MARPE tooth-bone-borne with bicortical anchorage. Excessive dental inclination related to the stress mechanism on the teeth could be avoided if the forces were applied directly to the bone with bicortical anchorage [29–31].

Celenk-Koca et al. [31], in an randomized clinical trials (RCT) considered a low risk of bias study, observed less buccal inclination in the first premolar and first molar, in a group of adolescent patients who received maxillary expansion with skeletal anchorage of four mini-implants compared to conventional RME.

The studies by Li et al. (MARPE tooth-bone-borne) [20], Lin et al. (MARPE bone-borne) [22] and Sygourus et al. (SARME tooth-borne with or without pterygoid disjunction) [23], reported a decrease in the height of the alveolar crest of less than − 0.7 mm. While Gauthier et al. (SARME tooth-borne) [24] reported a greater decrease of the alveolar crest of − 1.42 mm, without recording the number of daily activations of the expander. On the other hand, Pham and Lagravère [32], in adolescents, found no differences in the loss of marginal alveolar bone in the posterior tooth and therefore the results were not clinically significant. The results are not conclusive about which intervention had more or less of a decrease in alveolar crest height, however, the < 1 mm of bone loss, suggests that these changes would not be clinically significant.

The decrease in the buccal alveolar thickness is minimal using MARPE bone-borne (C-expander = − 0.07 mm) [19]. This behavior could be due to the fact that the C-expander does not incorporate bands in its clinical installation, unlike the MARPE tooth-bone-borne (MSE I = -0.58 mm) [19] or SARME [21, 23, 24] (hyrax tooth-borne = − 0.63 mm on average). Similarly, SARME tooth-bone-borne at the level of the first molar (hybrid hyrax = − 0.64) [21].

In the same way, Celenk-Koca et al. [31], reported a decrease in buccal alveolar thickness, showing a smaller decrease in alveolar bone in the group of adolescent patients treated with maxillary expansion with skeletal anchorage compared to the group with RME.

Kayalar et al. and Gauthier et al. reported a significant increase in palatal alveolar thickness, with an average less than 1 mm, using SARME, due to tooth movement generated by lateral forces transmitted to the teeth using SARME tooth borne [21, 24] or tooth-bone-borne [24].

Lin et al. [22], reported an average dehiscence of 0.22 mm, *P* < .05; and Moon et al. [19], reported an incidence of 31.3% (15/48 cases) with MSE and 4.2% (2/48 cases) with C-expander, with significant differences when comparing both groups.

Moon et al. [19], reported fenestrations in 6/48 cases with MSE I and 1/48 case with C-Expander, without significant differences when comparing both groups.

There is a lower incidence of bone defects in MARPE bone borne compared to MARPE tooth bone borne.

These bone defects are commonly documented as a result of RME due to osteoclastic activity of the teeth that move through the buccal alveolar bone [12, 33–35].

### Study considerations

The results of the present study are based on a limited number of studies, with limited evidence from randomized clinical trials regarding SARME and non-randomized clinical trials for MARPE.

All included studies have adequate designs and methodologies to evaluate treatment effectiveness (randomized trials and cohorts). In addition, they show a low risk of bias, which, although is not an overall assessment of methodological quality, strengthens the idea that their observations and results are reliable and allows authors to provide recommendations for guidelines.

The results presented focus on the immediate changes (3–6 months) after expansion, thus the next suggested step is to view results over longer periods of times to verify the increase, decrease or stability of these findings.

## Conclusions

1. Although limited, the current evidence seems to show that bone-borne or tooth-bone-borne with bicortical skeletal anchorage produces fewer undesirable periodontal effects, such as: alveolar bending, dental inclination and decrease of the alveolar crest on MARPE compared to tooth-borne or tooth-bone-borne on SARME.
2. The tooth-bone-borne monocortical skeletal anchorage on MARPE and tooth-borne or tooth-bone-borne on SARME, produce similar undesirable periodontal effects.
3. The tooth-bone-borne on SARME might be a beneficial alternative to reduce harmful periodontal effects compared to SARME tooth-borne.

## Supplementary Information

**Additional file 1.**

## Data Availability

The datasets used and/or analyzed during the current study are available from the corresponding author on reasonable request.

## Abbreviations

PubMed: Public Medline
MEDLINE: Medical Literature Analysis and Retrieval System Online
EMBASE: Excerpta Medica database
LILACS: Literatura latinoamericana y del Caribe en ciencias de la salud
ROB: Risk of bias
NOS: Newcastle-Ottawa Scale
RME: Non-surgical maxillary expansion
SARME: Surgical assisted rapid maxillary expansion
MARPE: Miniscrew-assisted rapid palatal expansion
PROSPERO: International database of prospectively registered systematic review in health
PRISMA: Preferred Reporting Items for Systematic Reviews and Meta-Analyses.
PICOT: Population, intervention, comparison, outcome and time.
MSE: Maxillary skeletal expander
HYRAX: Hygienic rapid expander
CBCT: Cone-beam computed tomography
