# Supplementary material for: Periodontal effects of maxillary expansion in adults using non-surgical expanders with skeletal anchorage vs. surgically assisted maxillary expansion: a systematic review
Source: Head Face Med. 2021 Nov 10;17:47. doi: 10.1186/s13005-021-00299-7 (PMC8579525; doi:10.1186/s13005-021-00299-7)
Supplement: Supplementary file 1 — Additional file 1. [file 13005_2021_299_MOESM1_ESM.docx]

**Appendix A**

**PUBMED**

(((((("palatal expansion technique"[MeSH Terms] OR (("palatal"[All Fields] AND "expansion"[All Fields]) AND "technique"[All Fields])) OR "palatal expansion technique"[All Fields]) OR ("maxillary"[All Fields] AND "expansion"[All Fields])) OR "maxillary expansion"[All Fields]) OR (((((((("palatalization"[All Fields] OR "palatalized"[All Fields]) OR "palatally"[All Fields]) OR "palatals"[All Fields]) OR "palate"[MeSH Terms]) OR "palate"[All Fields]) OR "palatal"[All Fields]) OR "palates"[All Fields]) AND ((((("expanse"[All Fields] OR "expanses"[All Fields]) OR "expansion"[All Fields]) OR "expansions"[All Fields]) OR "expansive"[All Fields]) OR "expansively"[All Fields]))) AND ("gingi*"[All Fields] OR "perio*"[All Fields])) AND ((("adult"[MeSH Terms] OR "adult"[All Fields]) OR "adults"[All Fields]) OR "adult s"[All Fields])

**MEDLINE**

**1 Maxillary Expansion.mp. (1550)**

**2 Palatal Expansion.mp. (3018)**

**3 1 or 2 (3336)**

**4 exp Adult/ or adult.mp. (7829646)**

**5 3 and 4 (776)**

**6 perio*.mp. (2038542)**

**7 gingi*.mp. (68256)**

**8 6 or 7 (2070682)**

**9 5 and 8 (185)**

**10 limit 9 to humans (180)**

**COCHRANE**

**ID Search Hits**

**#1 maxillary expansion 374**

**#2 palatal expansion 254**

**#3 #1 or #2 417**

**#4 adult 623162**

**#5 #3 and #4 77**

**LILACS**

**maxillary expansion OR palatal expansion [Words] and adult [Words]**
